# Natural selection drives rapid evolution of mouse embryonic heart enhancers

**DOI:** 10.1186/1752-0509-6-S2-S1

**Published:** 2012-12-12

**Authors:** Ben-Yang Liao, Meng-Pin Weng

**Affiliations:** 1 Division of Biostatistics & Bioinformatics, Institute of Population Health Sciences, National Health Research Institutes, Zhunan Town, Miaoli County 350, Taiwan, ROC

## Abstract

**Background:**

Mouse E11.5 embryonic heart enhancers were found to exhibit exceptionally weak sequence conservation during vertebrate evolution compared to enhancers of other developing organs. However, it is unknown whether this phenomenon is due to elevated mutation rates, or is a consequence of natural selection.

**Results:**

In this study, based on the aligned orthologous genomic sequences of mouse and other closely related mammals, the substitution rates of fourfold degenerate sites or intron sequences in neighboring genes were used as neutral references to normalize substitution rates of mouse enhancers. Subsequent comparisons indicated that heart enhancers' evolutionary rates were increased by natural selection. Correspondingly, the results of Fisher's exact tests to examine the differential enrichment of substitutions between enhancers and neutral sequences suggest that both relaxed purifying selection and positive selection caused the rapid evolution of heart enhancers. Analyses on recombination rates and substitution patterns indicated that GC-biased gene conversion does not contribute to evolutionary rate variations among enhancers. In general, pleiotropic enhancers and enhancers in proximity to weakly expressed genes, tend to evolve slowly. Although heart enhancers are less pleiotropic and are adjacent to highly expressed genes, these biases do not account for the rapid evolution observed.

**Conclusions:**

In combination, the results of the present study suggest that factors associated with functions or characteristics of the tissue may exert direct and profound effects on the intensity and direction of the natural selection applied to regulatory DNAs, such as enhancers.

## Background

Evolution of gene expression may underlie the morphological diversity of animal species [1-3]. Therefore, it is important to understand the rules that govern the evolution of regulatory DNA sequences. Enhancers are a major category of regulatory DNAs, and are short genomic segments that bind to regulatory proteins and increase gene transcription [4,5]. Correspondingly, enhancers have been shown to regulate many of the processes that affect organismal development [6,7]. To locate these important regulatory non-coding DNAs in the mouse (*Mus musculus*) genome, several thousand mouse enhancers that function in the embryonic heart, forebrain, midbrain, or limbs were recently identified using a p300 ChIP-Seq approach [8-10]. Subsequent analyses showed that the evolutionary signatures of the enhancers associated with different tissue types were variable: Embryonic heart enhancers exhibited an exceptionally low sequence conservation compared to enhancers from other tissues [9]. Since the heart is a highly conserved organ critical to the survival of all vertebrate organisms, DNAs associated with the regulatory mechanisms that control heart development were expected to be conserved across a long phylogenetic distance [11]. Although the underlying cause for the rapid sequence evolution observed in embryonic heart enhancers remains unknown, this unexpected phenomenon has challenged the use of sequence conservation as a criterion for predicting functionally important non-coding DNAs [12,13].

There are three potential explanations for the fast evolution rate observed for heart enhancers. First, the mutation rate of heart enhancers are elevated, similar to that of fast evolving DNAs that reside in genomic regions prone to replication errors [14-18]. Second, heart enhancers are subject to a more frequent, or intense, positive selection, similar to that of genes encoding adaptive proteins where new mutations that develop tend to be fixed more frequently [19,20]. Third, heart enhancers have evolved under a more relaxed purifying selection, similar to that of genes with non-essential functions that have high evolution rates relative to essential genes [21,22]. To examine if differential frequencies/intensities in mutation rate or natural selection have led to an increased evolution of heart enhancers, based on mouse-rat or mouse-human genomic alignments, substitution rates of fourfold degenerate sites (*d*_4_), or intron sites (*d*_i_), of neighboring genes were used as neutral references to normalize the substitution rate of each enhancer. Substitution rates of mouse enhancers from different tissues were then compared. Fisher's exact tests were also used to examine the differential enrichment of substitutions between enhancers and the corresponding neutral sequences, and these results were used to determine the strength of the purifying selection, or the frequency/intensity of the positive selection, experienced by the enhancers. We also examined the potential role for GC-biased gene conversion events in affecting the interpretation of processes that accelerate sequence substitutions of heart enhancers in mammals.

Studies on protein evolution have shown that unanticipated confounding factors could influence the identification and interpretation of previously reported protein evolutionary rate determinants. For instance, gene essentiality was asserted to influence bacterial protein evolutionary rates [23]. However, a later study found that this phenomenon was simply due to that highly expressed genes evolved slowly and essential genes were highly expressed [24]. Similarly, extracellularity was suggested to be correlated with the rate of protein evolution in yeasts [25]. Nevertheless, this was because extracellular proteins tend to be non-essential to the organism and non-essential genes were less constrained evolutionarily [26]. To determine whether the rapid evolution of heart enhancers is due to factors directly associated with functions or characteristics of the embryonic tissues or organs where a DNA performs functions (termed as "tissue factors" hereafter) [27,28], the influence of enhancer properties and neighboring genes to the evolution of these heart enhancers were examined. We found that the evolution rates of heart enhancers were correlated with pleiotropy (see Methods), as well as the expression levels of neighboring genes. Embryonic heart enhancers generally were less pleiotropic and were in proximity of highly expressed neighboring genes. After controlling for these biases, heart enhancers still exhibited highest rates of evolution. The results suggested that tissue factors may exert direct effects on the intensity and direction of the natural selection on enhancers. Further studies to elucidate how tissue factors specifically cause variations in selective forces on enhancers are needed.

## Methods

### Enhancers of developing mouse embryonic tissues

Based on the Ensembl mouse genome assembly v59 (NCBI m37), the co-activator protein, p300, was found to bind 2759, 2786, 3839, and 3597 enhancers in forebrain, midbrain, limb, and heart mouse embryonic tissues (Supplementary material in [9]). When enhancers with overlapping coordinates were consolidated into a single enhancer region, 11,332 non-overlapping enhancer regions were determined. These regions were further classified into "specific enhancers" (i.e., regions consolidated from enhancers of a single embryonic tissue) (n = 10,030) and "pleiotropic enhancers", the remaining set of enhancers (n = 1,302). It should be noted that, since only four tissues from the E11.5 stage were examined in p300 ChIP-seq experiments [9], the "specific enhancers" defined in the present study may function in other tissues or developmental stages during mouse embryogenesis. Furthermore, the term "pleiotropic" was used to describe enhancers that are likely to significantly affect the developmental processes of at least two of the examined embryonic organs in [9] when they are absent from the genome. The recombination rate of the mouse genome segment in which an enhancer was located was obtained from the Supplementary table of [29].

### Properties of the mouse genes

The coordinates of the mouse genes analyzed were retrieved from the BioMart (http://www.biomart.org/). For mouse genes with a null phenotype, the essentiality of each gene was defined based on phenotypic annotations of Mouse Genome Informatics 4.21 (http://www.informatics.jax.org/), according to [30]. When premature death or infertility were the knockout phenotype, these genes were considered essential. All other genes with at least one documented null phenotype were considered non-essential. The gene expression profiles for the mouse genes analyzed were obtained from microarray data collected using 61 mouse tissues, including 53 adult tissues, 3 cell lines, and 5 early embryos from E6.5 to E10.5 [31]. Expression levels were calculated by averaging microarray-based expression signals from all tissues according to the previous studies [22,26].

### Tests of natural selection on enhancer sequences

To study the patterns and determinants of enhancer evolution rates, aligned mouse-rat (*Rattus norvegicus*), mouse-human (*Homo sapiens*), and mouse-rat-human orthologous genomic regions were retrieved from Ensembl (http://www.ensembl.org/) [32], or the UCSC Genome Browser (http://genome.ucsc.edu) [33] (genome versions: mouse: mm9; rat: rn4; human: hg18). Sequence divergence was subsequently calculated, and the numbers of substitutions were computed. Based on the genomic alignments retrieved from the UCSC Genome Browser (http://genome.ucsc.edu) [33], the sequence divergence of enhancer regions (*D*) from mouse versus other mammal sequences was calculated according to *baseml *of PAML [34] using a GTR model with gamma distribution for site heterogeneity (model = 7; ncatG = 5). Fourfold degenerate sites and intron sequences of the nearest gene with an ortholog in the rat or human genome were used as neutral references to an enhancer. Since mammalian genes are often alternatively spliced and produce multiple transcripts [35], the coding sequence and intron sequence of a gene were defined using the exon-intron structure of the longest isoform annotated in Ensembl v59, with alignments of coding sequences and intron sequences retrieved from the UCSC Genome Browser. Fourfold degenerate sites of mouse genes were defined according to W-H Li [36]. When intron sequences were used as the neutral reference, similar to R Haygood, O Fedrigo, B Hanson, K-D Yokoyama and GA Wray [37], the first intron was removed due to the possibility it could contain regulatory motifs [38]. The latter criterion resulted in that intronless genes and single-intron genes have only the neutral reference of *d*_4_, and not *d*_i_. Similarly, sequence divergences between mouse and another mammal under neutrality, *d*_4 _and *d*_i_, were calculated by *baseml *of PAML [34] using a GTR model with gamma distribution for site heterogeneity (model = 7; ncatG = 5). As indicated above, the calculation of *d*_4 _or *d*_i _required the presence of an Ensembl annotation of a mouse gene to its one-to-one ortholog to ensure the reliability of neutral estimation. The normalized enhancer sequence divergences (*D*/*d*_4 _or *D*/*d*_i_) were then used as indexes for the relative strength of selection. Lower *D*/*d*_4 _or *D*/*d*_i _values indicated a weaker purifying selection, or a stronger positive selection, had occurred for the enhancer sequence examined [1].

According to AP Rooney and J Zhang [39], Fisher's exact test was used to examine the natural selection on enhancers based on the 2x2 contingency table. The row and column categories of the table are "the enhancer vs. the neutral reference used" and "the number of substituted sites vs. the number of sites with no substitution", respectively. Both fourfold degenerate sites, or intron sequences of the neighboring gene (defined above), were used as neutral references to examine the differential enrichment of substitutions between enhancers and neutral references. Compared to the neutral reference, significantly more substitutions in an enhancer implied that positive selection had occurred. Alternatively, fewer substitutions in an enhancer suggested that purifying selection had occurred. Otherwise, the model of neutrality cannot be rejected statistically, and the enhancer would be considered to have evolved free from natural selection.

## Results and discussion

### Natural selection leads to rapid evolution of embryonic heart enhancers

**Figure 1:**
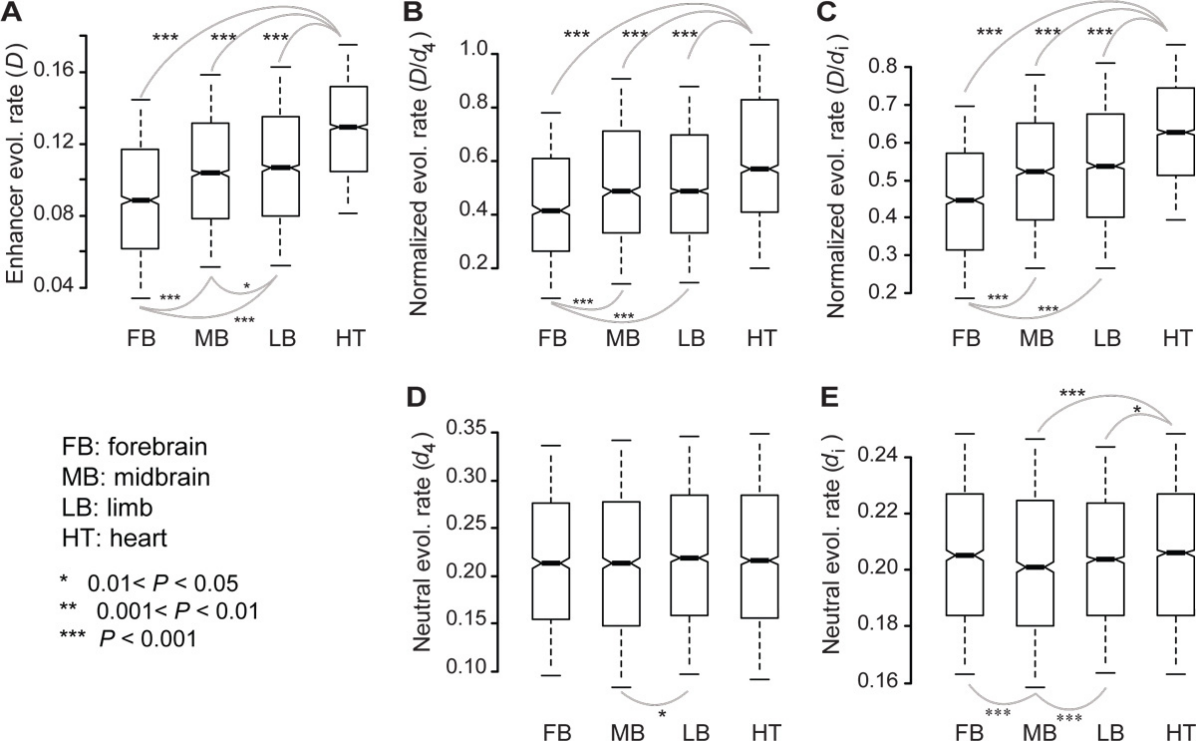
**Evolutionary rates of enhancers**. Rates of evolution calculated for mouse enhancers of embryonic forebrain (FB), midbrain (MB), limb (LM), and heart (HT), included *D *(**A**), normalized evolutionary rates *D*/*d*_4 _(**B**) or *D*/*d*_i _(**C**), and neutral substitution rates *d*_4 _(**D**) or *d*_i _(**E**). The values of upper quartile, median, and lower quartile are indicated in each box, whereas the bars outside the box indicate semi-quartile ranges. *D*, *D*/*d*_4_, *D*/*d*_i_, *d*_4 _and *d*_i _were computed based on mouse-rat alignments. Pairwise comparisons showing significant differences in *D*, *D*/*d*_4_, *D*/*d*_i_, *d*_4_, or *d*_i _are connected with gray lines (Mann-Whitney *U *test).

In a previous study, an analysis of conservation depth using DNA alignments from a wide range of vertebrates identified the rapid evolution of embryonic heart enhancers [9]. In the present study, sequence divergence, *D *(see Methods), was used to examine the evolution rate of enhancers, while *D*/*d*_4 _or *D*/*d*_i _(see Methods) ratios were used to examine the presence and direction of natural selection on non-coding sequences after the divergence of mouse-rat (Figure 1), or mouse-human (Additional file 1), genomes. Heart enhancers were found to have the highest *D *(indicating the weakest sequence conservation), while forebrain enhancers had the lowest *D *(indicating strongest sequence conservation) (mouse-rat: Figure 1A; mouse-human: Additional file 1A). These results are consistent with the order of enhancer types determined by the previous analysis of conservation depth for these tissues (Figure 1b of [9]). If the differences in *D *determined for enhancers of forebrain, midbrain, limb and heart tissues is only due to variations in local mutation rates, these differences should disappear when *D *is normalized to neutral substitution rates (e.g., approximately by *d*_4 _or *d*_i_). Based on the analysis performed, the orders of the enhancers of four tissue types in *D/d*_4 _(mouse-rat: Figure 1B; mouse-human: Additional file 1B) or *D/d*_i _(mouse-rat: Figure 1C; mouse-human: Additional file 1C) followed the order shown in *D*, while such an order was neither observed for *d*_4 _(mouse-rat: Figure 1D; mouse-human: Additional file 1D) nor *d*_i _(mouse-rat: Figure 1E; mouse-human: Additional file 1E). An estimation of mouse-rat *D*, *D/d*_4_, and *D/d*_i _with the inclusion of human orthologous sequence as the outgroup in the model of *baseml *yielded virtually identical results (Additional file 2). Taken together, these results clearly indicate that natural selection, and not mutation, is responsible for the rapid sequence divergence (e.g., large *D*) associated with heart enhancers.

The higher *D *of heart enhancers may be due to a more relaxed purifying selection, or to a more frequent and stronger positive selection, that occurred during evolution. To distinguish between these possibilities, Fisher's exact test [39] was used on each enhancer to examine differences in the enrichment of substitutions between enhancers and the neutral reference [i.e., fourfold degenerate sites present in the neighboring gene (Table 1 and Additional file 3) or intron sites in the neighboring gene (sites of the first intron were excluded, see Methods) (Table 2 and Additional file 4)]. If the enhancer evolved without the influence of natural selection, no significant difference in the proportion of nucleotides that underwent substitution after mouse-rat or mouse-human divergence between the enhancer and the neutral reference would be observed. Otherwise, enrichment or depletion of substitutions in the enhancer compared to the neutral reference would suggest that the enhancer had undergone positive selection or purifying selection, respectively. To minimize the effect of enhancer pleiotropy (see Methods, and below), only "specific enhancers" (see Methods) with available neutral references were used in the analysis. The sample size of the enhancers tested for Table 2 (or Additional file 4) was smaller than that of Table 1 (or Additional file 4) since enhancers with a nearby gene that did not have introns or single-exon genes were excluded due to the lack of neutral intron sites. Analyses of both mouse-rat alignments (Table 1, 2) and mouse-human alignments (Additional file 3, 4) were performed. Several consistent observations were made. First, in comparison with enhancers of the forebrain, midbrain, and limb, a smaller proportion of heart enhancers were subject to natural selection (see item "Under selection/Total" in Table 1, 2 and Additional file 3, 4). These results indicate that heart enhancers have undergone a more relaxed purifying selection. Second, a larger proportion of heart enhancers had higher substitution rates than the neutral reference, compared to the enhancers of other tissues (see items "Positively selected/Under selection" and "Positively selected/Total" in Table 1, 2 and Additional file 3, 4). These results indicate that mouse embryonic heart enhancers experienced more frequent or more intense, positive selections during their evolution.

**Table 1:** The proportions of enhancers that underwent selection based on mouse-rat alignments (neutral reference: fourfold degenerate sites)

	Total	Under selection^a^	Positively selected^b^	Under selection^a^/Total	Positively selected^b^/Under selection^a^	Positively selected^b^/Total
**HT**	2554	875	360	34.25%	41.14%	14.09%
**FB**	1253	595	67	47.48%	11.26%	5.34%
**MB**	1311	521	101	39.74%	19.38%	7.70%
**LB**	2570	1037	212	40.35%	20.44%	8.24%

FB: forebrain; MB: midbrain; LB: limb; HT: heart.

^a^A significantly unequal proportion of substituted sites were observed between enhancers and the fourfold degenerate sites of the neighboring gene by Fisher's exact test.

^b^Enhancers that underwent selection and had a higher substitution rate than the fourfold degenerate sites of the neighboring gene.

**Table 2:** The proportions of enhancers that underwent selection based on mouse-rat alignments (neutral reference: intron sites)

	Total	Under selection^a^	Positively selected^b^	Under selection^a^/Total	Positively selected^b^/Under selection^a^	Positively selected^b^/Total
**HT**	2475	1222	428	49.37%	35.02%	17.29%
**FB**	1159	748	83	64.53%	11.09%	7.16%
**MB**	1218	720	147	59.11%	20.41%	12.06%
**LB**	2470	1449	301	58.66%	20.77%	12.18%

FB: forebrain; MB: midbrain; LB: limb; HT: heart.

^a^A significantly unequal proportion of substituted sites were observed between enhancers and the intron sites of the neighboring gene by Fisher's exact test.

^b^Enhancers that underwent selection and had a higher substitution rate than the intron sites of the neighboring gene.

It is important to note that estimates of the proportion of enhancers under selection were subject to the neutral references used. For example, when intron sites were served as the neutral reference, a higher proportion of enhancers were found to undergo natural selection (item "Under selection/Total"). In addition, "Positively selected/Under selection" and "Positively selected/Total" values were found to slightly differ. There are several possible explanations for these observations. First, the available numbers and samples of enhancers used to generate Table 1 (or Additional file 3) and Table 2 (or Additional file 4) varied. Second, an enhancer's neighboring gene generally had a larger number of intron sites than fourfold degenerate sites for Fisher's exact tests. With an increase in neutral reference sites, a statistically significant result to reject the neutral model was more likely to be obtained. Although our estimations of "Under selection/Total", "Positively selected/Under selection", and "Positively selected/Total" values may be affected by changes in the neutral reference used, these changes were small, especially for results based on mouse-human alignments (Additional file 3 and 4). In addition, it should be noted that the present study focused on the relative values for "Under selection/Total", "Positively selected/Under selection", and "Positively selected/Total", not the absolute values, to compare enhancers from different tissues. Estimations of these three indexes using a similar approach and neutral references should be subjected to the same biases. The consistent patterns observed for data in Tables 1 and 2 and Additional files 3 and 4 unambiguously indicate that a higher tendency of being targeted by relaxed purifying selection or positive selection was associated with the more rapid evolution of mouse embryonic heart enhancers.

### GC-biased gene conversion does not explain the rapid evolution of heart enhancers

**Figure 2:**
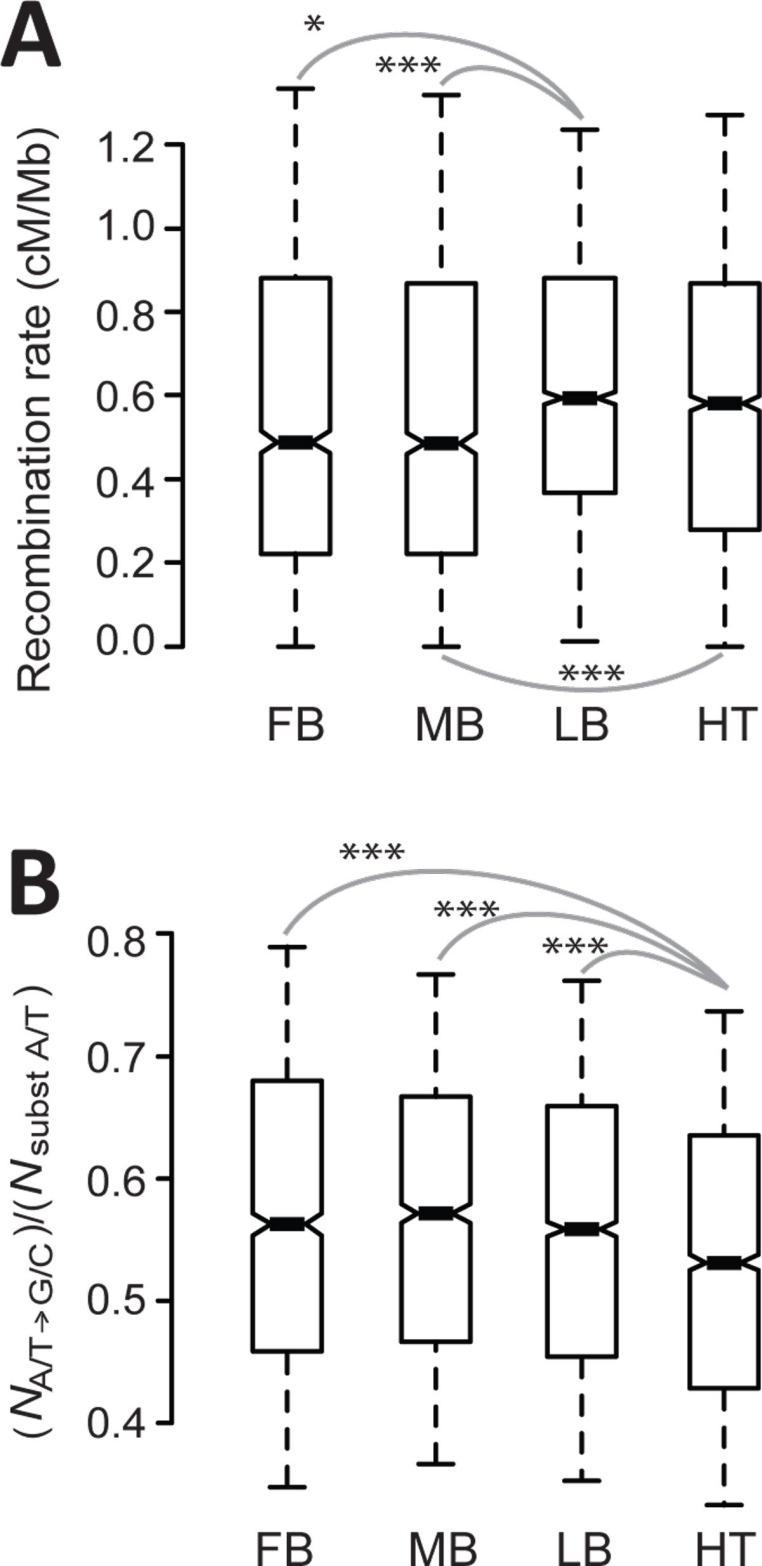
**Compared to other enhancers, heart enhancers are not subject to more frequent GC-biased gene conversions**. Heart enhancers (**A**) are not preferentially localized to genomic regions with higher recombination rates and (**B**) do not contain more A/T to G/C substitutions in the mouse lineages. The values of upper quartile, median, and lower quartile are indicated in each box, whereas the bars outside the box indicate semi-quartile ranges. Pairwise comparisons showing significant differences are connected with gray lines (*0.01<*P*≦0.05 and ***P≦0.001 by Mann-Whitney *U *test). FB, forebrain; MB, midbrain; LB, limb; HT, heart.

GC-biased gene conversion can mimic positive selection by generating similar genomic patterns, thereby confounding inferences regarding the type of selection that has occurred (reviewed in [40]). To investigate the possibility that a GC-biased gene conversion pattern could be misinterpreted as a positive selection of heart enhancers, we examined whether heart enhancers are located on genomic regions with higher recombination rates (recombination involves the formation of heteroduplex DNA that triggers gene conversion). Using the most recent recombination map of the mouse genome [29], although genomic regions containing heart enhancers were found to have relatively high recombination rates, these recombination rates were not higher than those of the genomic regions containing limb enhancers (Figure 2A) (the *D*, *D*/*d*_4_, and *D*/*d*_i _for heart enhancers were higher than those of limb enhancers, see Figure 1, A-C).

Furthermore, based on alignments of mouse-rat-human orthologous DNAs, the number of nucleotides that were ancestrally A or T before mouse-rat divergence (human and rat are both either A or T of the aligned site) and had a substitution event in the mouse lineage was calculated and defined as "*N*_substituted __A/T_" for each enhancer. The number of mouse lineage-specific substitutions from A/T to G/C (*N*_A/T→G/C_) was also calculated for each enhancer. If heart enhancers underwent more GC-biased gene conversion events, they would be predicted to have a higher *N*_A/T→G/C _to *N*_substituted __A/T _ratio. However, heart enhancers were associated with a significantly lower ratio of *N*_A/T→G/C _to *N*_substituted __AT _(Figure 2B) compared to the enhancers of other tissues.

In combination, the results shown in Figure 2 indicate that heart enhancers did not experience more GC-biased gene conversion events over their evolution. Therefore, mechanisms associated with GC-biased gene conversion did not explain the rapid evolution of heart enhancers and the selective pressure acting on them.

### Determinants for the evolution rate of enhancer sequences

**Figure 3:**
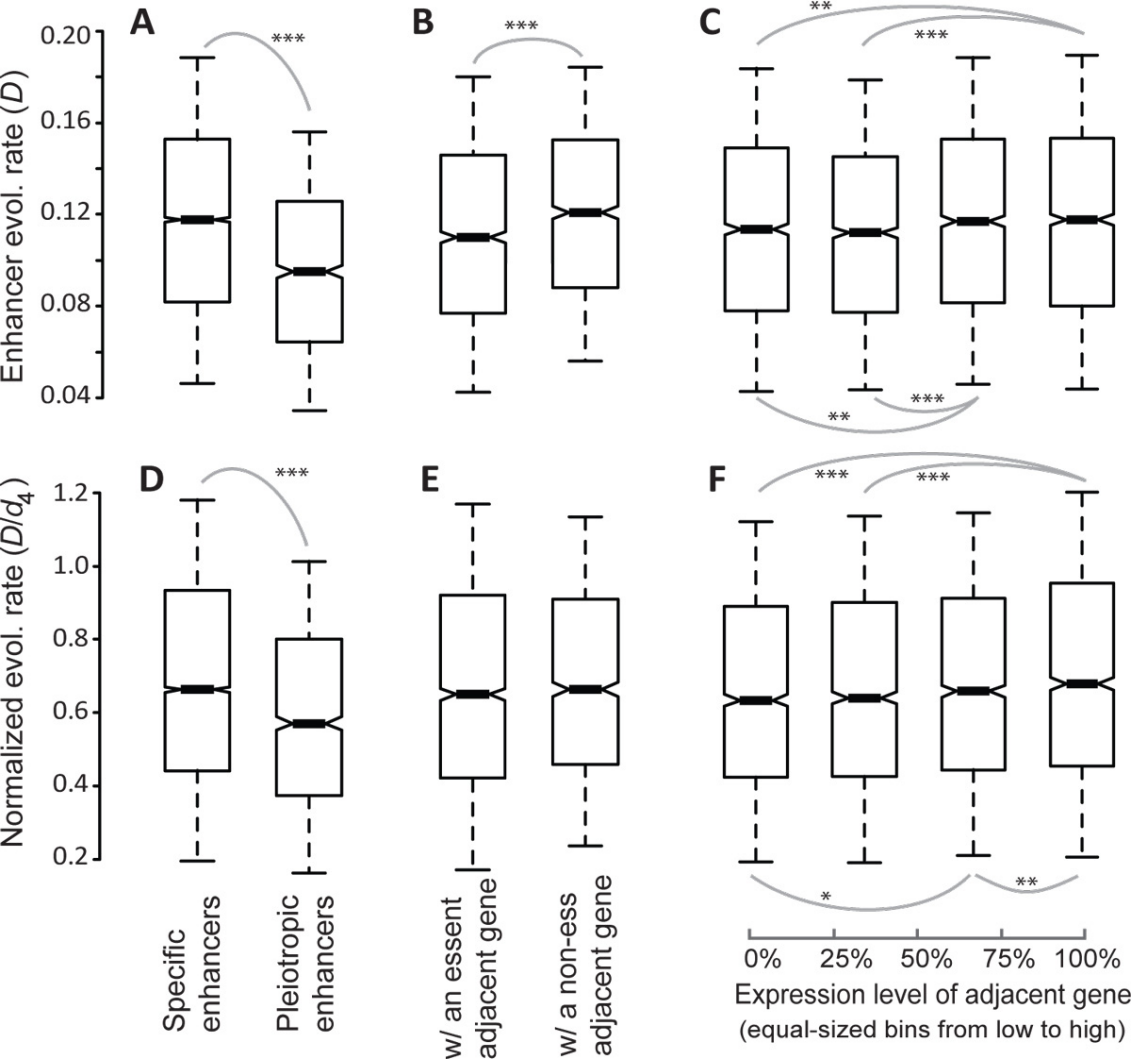
**Correlations between the evolutionary rates of mouse enhancers and enhancer properties: the tissue-specificity of the enhancers (**A, D**), essentiality of the adjacent gene (**B, E**), and the expression level of the adjacent gene (**C, F**)**. Evolutionary rates are represented by *D *(**A-C**) or *D*/*d*_4 _(**D-F**). Spearman's rank correlation coefficient is (**C**) 0.040 (*P *<10^-3^) and (**F**) 0.046 (*P *<10^-4^) for the unbinned data. The values of upper quartile, median, and lower quartile are indicated in each box, whereas the bars outside the box indicate semi-quartile ranges. Pairwise comparisons showing significant differences in *D *or *D*/*d*_4 _are connected with gray lines (*0.01<*P*≦0.05 and ***P≦0.001 by Mann-Whitney *U *test).

To understand the source of the selective forces acting on heart enhancers, the factors potentially associated with the evolution rate of enhancer sequences, and the properties of heart enhancers, were investigated. Two types of enhancer characteristics were examined: enhancer pleiotropy and the properties of neighboring genes. As mentioned in Methods, enhancer regions that only functioned in a single tissue were defined as specific enhancer regions, while all others were defined as pleiotropic enhancer regions. Initially, specific enhancer regions were found to have a significantly higher *D *(*P *< e-300, Mann-Whitney *U *test, Figure 3A) compared to pleiotropic enhancer regions. These results indicated that specific enhancer regions had a faster rate of sequence evolution. Furthermore, higher *D*/*d*_4 _(*P *= 6.7e-16, *U *test, Figure 3D) ratios of specific enhancer regions suggests that the faster evolution rate observed is due to a fixation bias, rather than a mutation bias. To avoid redundancy, analyses based on *D*/*d*_i _were not conducted. Previous studies reported that pleiotropic [41], or housekeeping protein coding genes [42], are subject to more stringent selective constraints. Similarly, the evolution of pleiotropic enhancer regions could be constrained by having a role in the development of a wide range of biological systems. As a result, mutations in these enhancer regions would have greater deleterious effects. These hypotheses are consistent with the patterns observed in Figure 3A and 3D.

Enhancers tend to regulate neighboring genes that are within several kilobases [9]. Therefore, we also investigated the association between the evolution rate of enhancer regions and the properties of adjacent genes (e.g., the closest coding gene in the mouse genome). The median distance for adjacent genes was found to be 49.7 kb. It is important to note that the adjacent gene of an enhancer may not be the same gene used to compute *d*_4 _or *d*_i_, since the calculation of *d*_4 _and *d*_i _require the presence of a one-to-one ortholog in another genome. For enhancer regions with an adjacent gene that is considered to be essential, a significantly smaller *D *(*P *= 3.83e-6, *U *test) was observed compared to enhancer regions adjacent to a non-essential gene (Figure 3B). These results suggest that faster rates of evolution occurred in the latter case. It has previously been shown that genomic regions with high gene density have low mutation rates [43]. Moreover, highly expressed genes, which tend to be essential [24,44], are located in gene-dense genomic regions [45]. Thus, we expected that genomic regions enriched with essential genes would have a lowered mutation rate which potentially explains Figure 3B. As expected, a smaller *D *for enhancer regions with an adjacent gene that is essential was observed, while no significant difference in *D*/*d*_4 _was observed for enhancers with an adjacent essential gene versus a non-essential gene (*P *= 0.27, *U *test, Figure 3E).

In addition, enhancer regions with a highly expressed adjacent gene tended to evolve faster than enhancer regions adjacent to a weakly expressed gene (Spearman's rank correlation coefficient σ of *D *vs. the expression level of the adjacent gene = 0.040, *P *= 1.9e-4; Figure 3C, for *U *test). A slightly more significant positive correlation was found between *D*/*d*_4 _and the expression level of an adjacent gene (Spearman's σ = 0.044, *P *= 9.8e-5; Figure 3F, for *U *test), suggesting that embryonic enhancers near weakly expressed genes are subject to a more biased selective force. The results remained unchanged when gene expression levels were defined by the maximum expression signal detected for the 61 mouse tissues analyzed (Additional file 5). However, at the present stage, it remains unclear whether expression levels of an adjacent gene directly, or indirectly, affect *D*/*d*_4_. Determining the cause of such an effect needs future studies.

### Local genomic and regulatory characteristics do not explain the rapid evolution of heart enhancers

**Figure 4:**
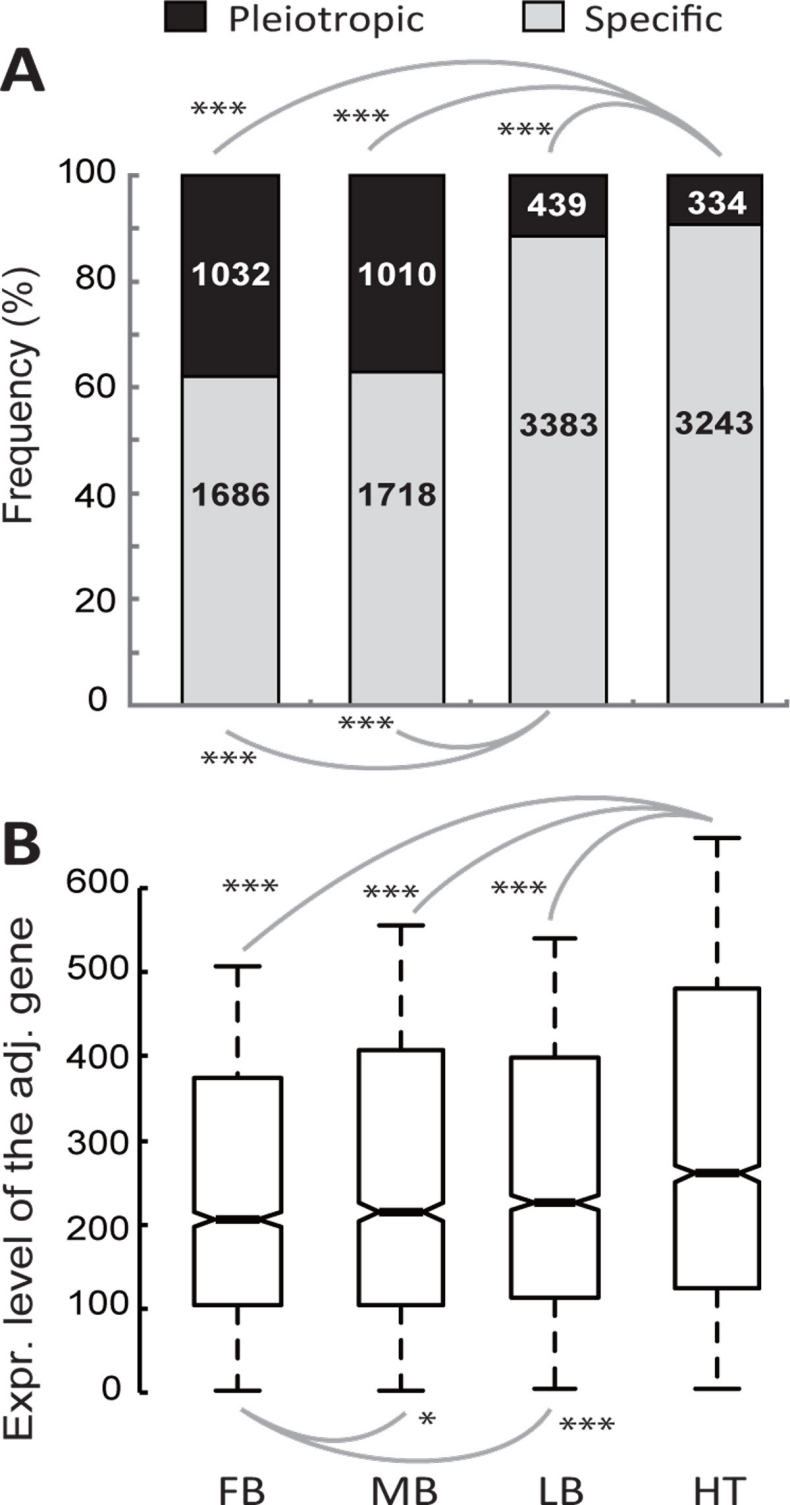
**Properties of heart enhancers**. Heart enhancers include more specific enhancers (**A**) and enhancers adjacent to highly expressed genes (**B**). (**A**) The numbers in boxes represent the number of enhancers in a group. (**B**) The values of upper quartile, median, and lower quartile are indicated in each box, whereas the bars outside the box indicate semi-quartile ranges. Pairwise comparisons with significantly different expression levels of the adjacent gene are connected with gray lines (*0.01<*P*≦0.05 and ***P≦0.001 by (**A**) × ^2 ^test or (**B**) Mann-Whitney *U *test). FB, forebrain; MB, midbrain; LB, limb; HT, heart.

As described in the Introduction, studies of protein evolution have shown that unanticipated confounding factors can influence the identification and the interpretation of determinants for coding DNA evolutionary rates. Therefore, variations in the evolution rates of different embryonic tissues can result from factors indirectly related to the tissue factor. In the present study, enhancers from the forebrain, midbrain, limb, and heart did not only differ in the tissue types from which they were identified, but also in several of their general properties that influenced *D *as well as *D*/*d*_4_. For embryonic heart enhancers, they typically were not pleiotropic (Figure 4A), and they tended to be physically close to highly expressed genes (Figure 4B). Since both tissue-specific enhancers and enhancers near highly expressed genes have been associated with fast evolution rates due to natural selection (Figure 3D and 3F), these biases could also contribute to the rapid evolution of embryonic heart enhancers.

**Figure 5:**
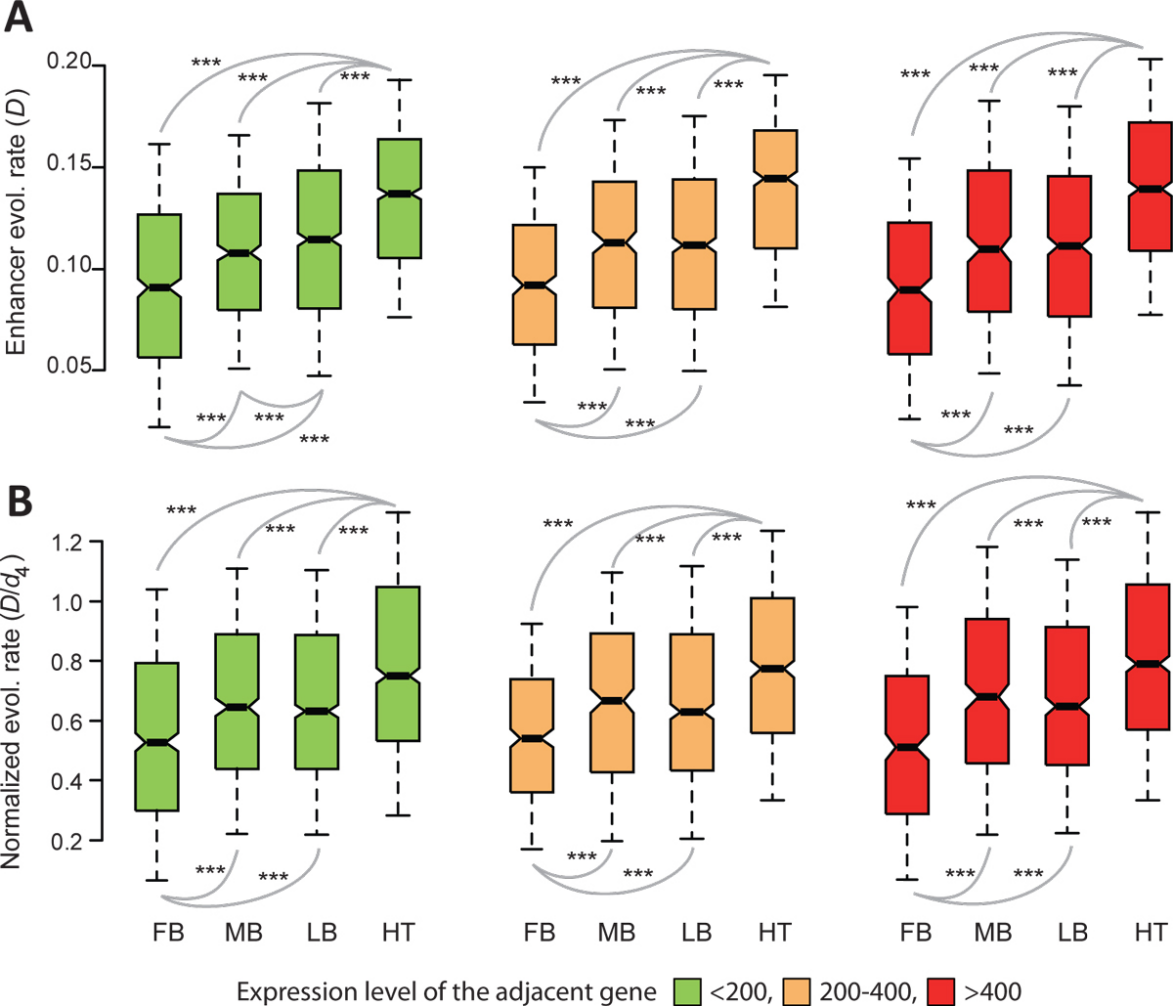
**Pleiotropy and expression levels of adjacent genes do not explain enhancer evolutionary rate variations**. Comparisons of *D *(**A**) and *D*/*d*_4 _(**B**) for mouse forebrain (FB), midbrain (MB), limb (LM), and heart (HT) enhancers after controlling for the enhancer pleiotropy and the expression level of adjacent genes. Only specific enhancers are included in the analysis. The values of upper quartile, median, and lower quartile are indicated in each box, whereas the bars outside the box indicate semi-quartile ranges. Pairwise comparisons showing significant difference in *D *or *D*/*d*_4 _are connected with gray lines (***P≦0.001 by Mann-Whitney *U *test).

To determine the role of enhancer pleiotropy and proximity to highly expressed genes, *D *(Figure 4A) and *D*/*d*_4 _(Figure 4B) values were compared for enhancers of the embryonic forebrain, midbrain, limbs, and heart, after controlling for the aforementioned two biases. To control for tissue specificity, only specific enhancers were examined. To control for the expression level of adjacent genes, enhancers were divided into three groups depending on the expression level of the adjacent gene, < 200, 200-400, and ≥ 400. *D *and *D*/*d*_4 _were then compared for the enhancers of the four tissues within a given group. After such controls, embryonic heart enhancers still had the highest *D *(Figure 5A) and *D*/*d*_4 _(Figure 5B) values. These results suggest that the relatively low pleiotropy of embryonic heart enhancers, and their physical proximity to highly expressed genes, only partially accounts for their high rate of evolution and biases in selection.

## Conclusions

The rapid evolution rates of mouse embryonic heart enhancers can potentially be explained by elevated rates of mutation and/or changes in the direction or intensity of natural selection. In the present study, neutral sites of the neighboring gene were used as references to examine mutation bias, as well as selective bias, on the evolution of enhancer sequences. Subsequent analyses demonstrated that the rapid evolution of mouse E11.5 heart enhancers cannot be explained by potential confounding factors examined in the present study, and therefore, may be directly associated with embryonic tissue factors.

The identification and understanding of regulatory DNAs in the mammalian genome is a major goal in the post-genomic era. This study employed a simple, yet effective, method to explore the role of natural selection on the evolution of non-coding sequences. Although the present study indicates that the rapid evolution of heart enhancers is likely to have been the result of a selective force associated with the type of embryonic tissue involved, the underlying causes for the relaxed selective constraint, or the higher frequency/intensity of positive selection, remain to be discovered. One possible underlying cause for a more relaxed purifying selection on heart enhancer is that during mouse embryogenesis, heart development begins and finishes earlier than brain and limb developments. If all enhancer sequences identified in [9] are functional regulatory DNA sequences at E11.5, these enhancers are regulating late-stage heart organogenesis but early-stage organogenesis of the brain or limbs. Mutations resulting in abnormal organ development at a later stage of organogenesis could bring a milder defect than those resulting in defects occur at an earlier stage, and therefore are more tolerable to the organism.

Our finding that heart enhancers tend to be positively selected is particularly intriguingly. In the future, it would be interesting to determine whether heart enhancers have been repeatedly shaped by positive selection because they are also involved in other developmental processes associated with morphological or physiological diversity. With the increasing abundance of functional genomic data and phenotypic data for mouse genes, such investigations will be feasible when a more complete set of spatial and temporal p300 ChIP-Seq data for mouse embryos and other species becomes available.

## List of abbreviations

*d*_4_: substitution rate of fourfold degenerate sites; *d*_i_: substitution rate of intron sites; *D*: sequence divergence of enhancer regions; FB: forebrain; MB: midbrain; LB: limb; HT: heart.

## Competing interests

The authors declare that they have no competing interests.

## Authors' contributions

B.-Y.L. designed research; B.-Y.L. and M.-P.W. performed research; M.-P.W. analyzed data; B.-Y.L. wrote the paper. All authors read and approved the final manuscript.

## Supplementary Material

Additional file 1 **Supplementary figure S1**. Evolutionary rates of enhancers. Rates of evolution calculated for mouse enhancers of embryonic forebrain (FB), midbrain (MB), limb (LM), and heart (HT), included *D *(**A**), normalized evolutionary rates *D*/*d*_4 _(**B**) or *D*/*d*_i _(**C**), and neutral substitution rates *d*_4 _(**D**) or *d*_i _(**E**). The values of upper quartile, median, and lower quartile are indicated in each box, whereas the bars outside the box indicate semi-quartile ranges. *D*, *D*/*d*_4_, *D*/*d*_i_, *d*_4 _and *d*_i _were computed based on mouse-human alignments. Pairwise comparisons showing significant differences in *D*, *D*/*d*_4_, *D*/*d*_i_, *d*_4_, or *d*_i _are connected with gray lines (Mann-Whitney *U *test). Click here for file

Additional file 2 **Supplementary figure S2**. Evolutionary rates of enhancers. Rates of evolution calculated for mouse enhancers of embryonic forebrain (FB), midbrain (MB), limb (LM), and heart (HT), included *D *(**A**), normalized evolutionary rates *D*/*d*_4 _(**B**) or *D*/*d*_i _(**C**), and neutral substitution rates *d*_4 _(**D**) or *d*_i _(**E**). The values of upper quartile, median, and lower quartile are indicated in each box, whereas the bars outside the box indicate semi-quartile ranges. *D*, *D*/*d*_4_, *D*/*d*_i_, *d*_4 _and *d*_i _were computed based on mouse-rat-human multiple alignments. Pairwise comparisons showing significant differences in *D*, *D*/*d*_4_, *D*/*d*_i_, *d*_4_, or *d*_i _are connected with gray lines (Mann-Whitney *U *test). Click here for file

Additional file 3 **Supplementary table S1**. The proportions of enhancers that underwent selection based on mouse-human alignments (neutral reference: fourfold degenerate sites) Click here for file

Additional file 4 **Supplementary table S2**. The proportions of enhancers that underwent selection based on mouse-human alignments (neutral reference: intron sites) Click here for file

Additional file 5 **Supplementary figure S3**. Enhancers with a highly expressed adjacent gene evolve more rapidly. Evolutionary rates are represented by *D *(**A**) or *D*/*d*_4 _(B). Gene expression level was defined by the maximum expression signal of the 61 mouse tissues. Spearman's rank correlation coefficient is (**C**) 0.044 (*P *<10^-4^) and (**F**) 0.042 (*P *<10^-3^) for the unbinned data. The values of upper quartile, median, and lower quartile are indicated in each box, whereas the bars outside the box indicate semi-quartile ranges. Pairwise comparisons showing significant differences in *D *or *D*/*d*_4 _are connected with gray lines (*0.01<*P*≦0.05 and ***P≦0.001 by Mann-Whitney *U *test). Click here for file
