# Supplementary material for: Natural selection drives rapid evolution of mouse embryonic heart enhancers
Source: BMC Syst Biol. 2012 Dec 12;6(Suppl 2):S1. doi: 10.1186/1752-0509-6-S2-S1 (PMC3521173; doi:10.1186/1752-0509-6-S2-S1)
Supplement: Additional file 1 — Supplementary figure S1. Evolutionary rates of enhancers. Rates of evolution calculated for mouse enhancers of embryonic forebrain (FB), midbrain (MB), limb (LM), and heart (HT), included D (A), normalized evolutionary rates D/d4 (B) or D/di (C), and neutral substitution rates d4 (D) or di (E). The values of upper quartile, median, and lower quartile are indicated in each box, whereas the bars outside the box indicate semi-quartile ranges. D, D/d4, D/di, d4 and di were computed based on mouse-human alignments. Pairwise comparisons showing significant differences in D, D/d4, D/di, d4, or di are connected with gray lines (Mann-Whitney U test). [file 1752-0509-6-S2-S1-S1.pdf]

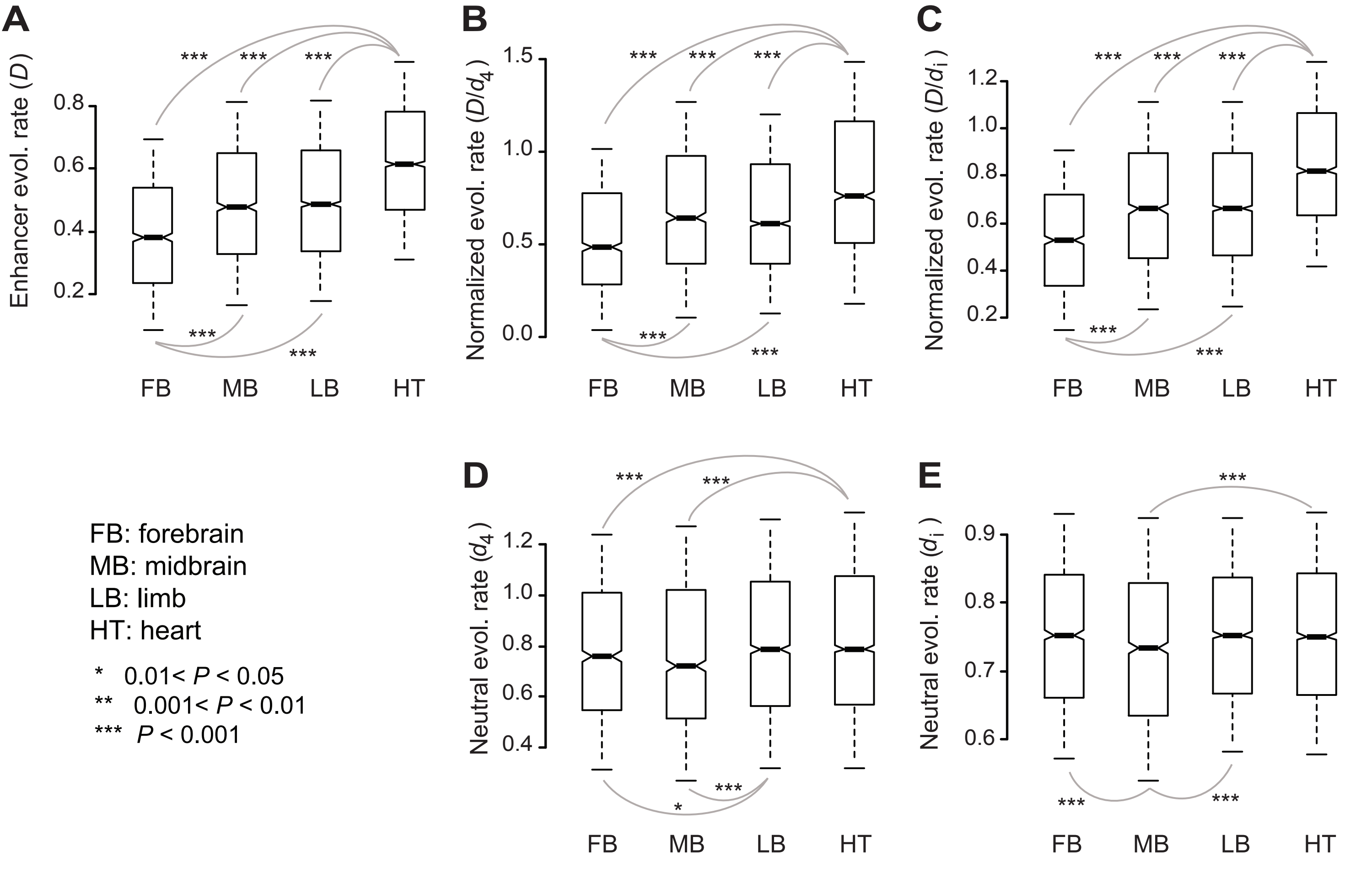

A
Enhancer evol. rate (D)
0.2
0.4
0.6
0.8
***
***
***
***
***
FB
MB
LB
HT
B
Normalized evol. rate (D/d4)
0.0
0.5
1.0
1.5
***
***
***
***
***
FB
MB
LB
HT
C
Normalized evol. rate (D/di)
0.2
0.4
0.6
0.8
1.0
1.2
***
***
***
***
***
FB
MB
LB
HT
D
Neutral evol. rate (d4)
0.4
0.6
0.8
1.0
1.2
***
***
***
*
FB
MB
LB
HT
E
Neutral evol. rate (di)
0.6
0.7
0.8
0.9
***
***
***
FB
MB
LB
HT
FB: forebrain
MB: midbrain
LB: limb
HT: heart
* 0.01< P < 0.05
** 0.001< P < 0.01
*** P < 0.001
