# Supplementary figures and images for: Natural selection drives rapid evolution of mouse embryonic heart enhancers
Source: BMC Syst Biol. 2012 Dec 12;6(Suppl 2):S1. doi: 10.1186/1752-0509-6-S2-S1 (PMC3521173; doi:10.1186/1752-0509-6-S2-S1)

**A**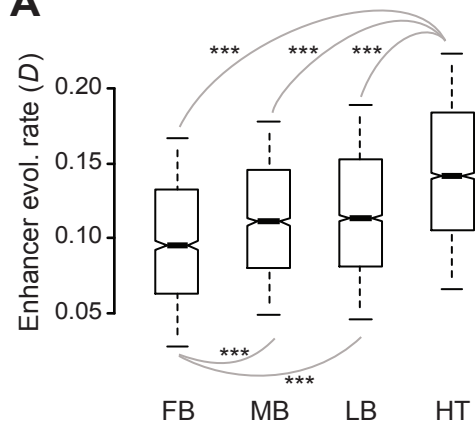**B**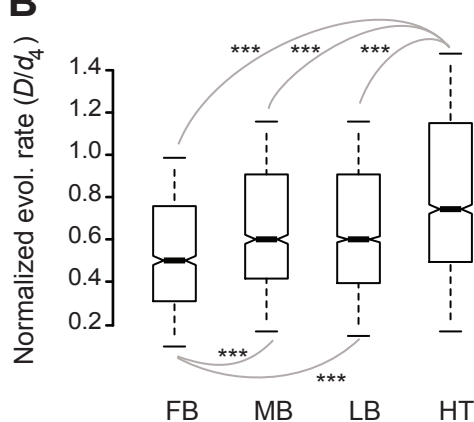**C**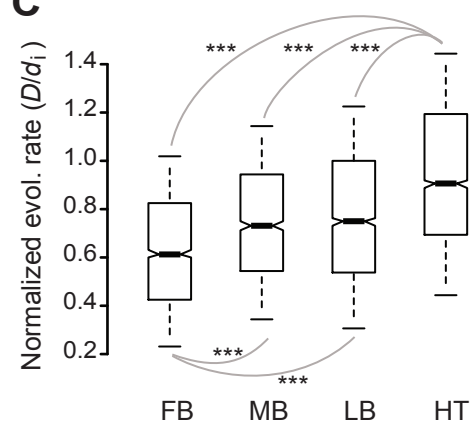

FB: forebrain  
 MB: midbrain  
 LB: limb  
 HT: heart

\*  $0.01 < P < 0.05$

\*\*  $0.001 < P < 0.01$

\*\*\*  $P < 0.001$

**D**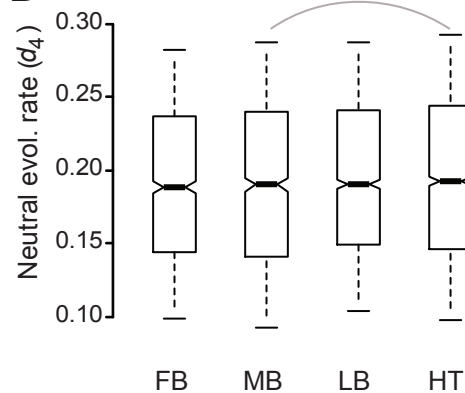**E**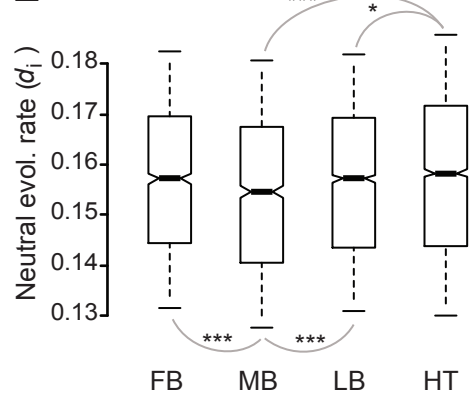

Supplement: Additional file 2 — Supplementary figure S2. Evolutionary rates of enhancers. Rates of evolution calculated for mouse enhancers of embryonic forebrain (FB), midbrain (MB), limb (LM), and heart (HT), included D (A), normalized evolutionary rates D/d4 (B) or D/di (C), and neutral substitution rates d4 (D) or di (E). The values of upper quartile, median, and lower quartile are indicated in each box, whereas the bars outside the box indicate semi-quartile ranges. D, D/d4, D/di, d4 and di were computed based on mouse-rat-human multiple alignments. Pairwise comparisons showing significant differences in D, D/d4, D/di, d4, or di are connected with gray lines (Mann-Whitney U test). [file 1752-0509-6-S2-S1-S2.pdf]

**A**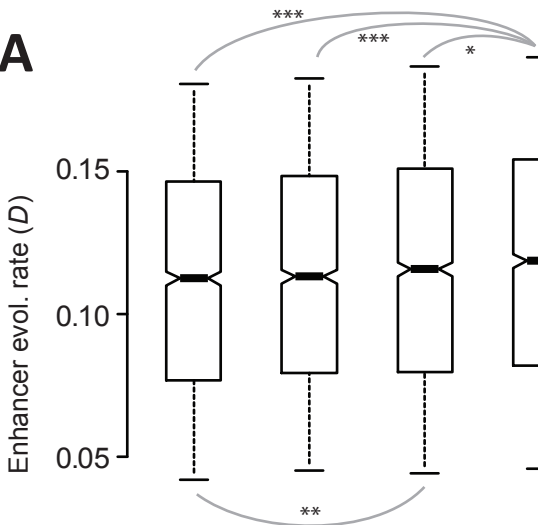**B**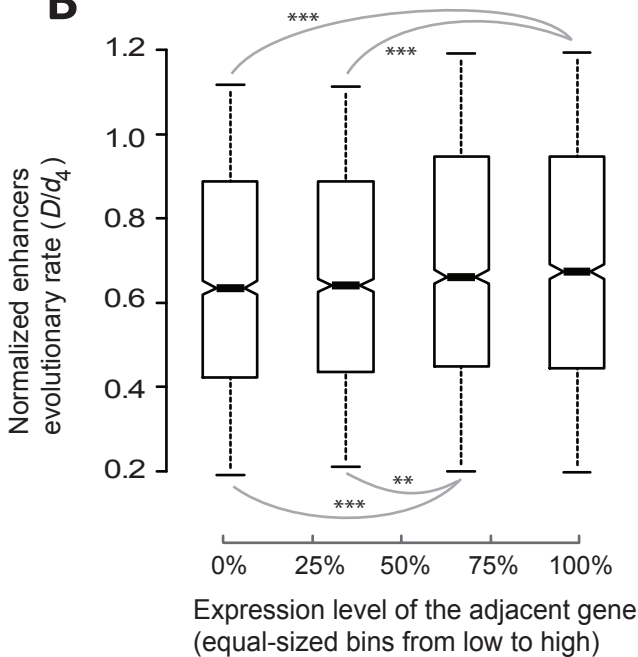

Supplement: Additional file 5 — Supplementary figure S3. Enhancers with a highly expressed adjacent gene evolve more rapidly. Evolutionary rates are represented by D (A) or D/d4 (B). Gene expression level was defined by the maximum expression signal of the 61 mouse tissues. Spearman's rank correlation coefficient is (C) 0.044 (P <10-4) and (F) 0.042 (P <10-3) for the unbinned data. The values of upper quartile, median, and lower quartile are indicated in each box, whereas the bars outside the box indicate semi-quartile ranges. Pairwise comparisons showing significant differences in D or D/d4 are connected with gray lines (*0.01<P≦0.05 and ***P≦0.001 by Mann-Whitney U test). [file 1752-0509-6-S2-S1-S5.pdf]
