# Supplementary material for: Natural selection drives rapid evolution of mouse embryonic heart enhancers
Source: BMC Syst Biol. 2012 Dec 12;6(Suppl 2):S1. doi: 10.1186/1752-0509-6-S2-S1 (PMC3521173; doi:10.1186/1752-0509-6-S2-S1)
Supplement: Additional file 3 — Supplementary table S1. The proportions of enhancers that underwent selection based on mouse-human alignments (neutral reference: fourfold degenerate sites) [file 1752-0509-6-S2-S1-S3.pdf]

Table S1. The proportions of enhancers that underwent selection based on mouse-human alignments (neutral reference: fourfold degenerate sites)

	**Total**	**Under selection**[a]	**Positively selected**[b]	**Under selection**[a] **/Total**	**Positively selected**[b] **/Under selection**[a]	**Positively selected**[b] **/Total**
**HT**	2316	915	428	39.50%	46.77%	18.48%
**FB**	1249	716	80	57.32%	11.17%	6.40%
**MB**	1269	598	123	47.12%	20.56%	9.69%
**LB**	2444	1166	282	47.70%	24.18%	11.53%

FB: forebrain; MB: midbrain; LB: limb; HT: heart.

[a] A significantly unequal proportion of substituted sites were observed between enhancers and the fourfold degenerate sites of the neighboring gene by Fisher's exact test.

[b] Enhancers that underwent selection and had a higher substitution rate than the fourfold degenerate sites of the neighboring gene.
