# Supplementary material for: Natural selection drives rapid evolution of mouse embryonic heart enhancers
Source: BMC Syst Biol. 2012 Dec 12;6(Suppl 2):S1. doi: 10.1186/1752-0509-6-S2-S1 (PMC3521173; doi:10.1186/1752-0509-6-S2-S1)
Supplement: Additional file 4 — Supplementary table S2. The proportions of enhancers that underwent selection based on mouse-human alignments (neutral reference: intron sites) [file 1752-0509-6-S2-S1-S4.pdf]

Table S2. The proportions of enhancers that underwent selection based on mouse-human alignments (neutral reference: intron sites)

	Total	Under selection[a]	Positively selected[b]	Under selection[a] /Total	Positively selected[b] /Under selection[a]	Positively selected[b] /Total
**HT**	2273	946	415	41.61%	43.86%	18.25%
**FB**	1183	724	79	61.20%	10.91%	6.67%
**MB**	1186	656	142	55.31%	21.64%	11.97%
**LB**	2368	1257	274	53.08%	21.79%	11.57%

FB: forebrain; MB: midbrain; LB: limb; HT: heart.

[a] A significantly unequal proportion of substituted sites were observed between enhancers and the intron sites of the neighboring gene by Fisher’s exact test.

[b] Enhancers that underwent selection and had a higher substitution rate than the intron sites of the neighboring gene.
